# RNA-Seq reveals 10 novel promising candidate genes affecting milk protein concentration in the Chinese Holstein population

**DOI:** 10.1038/srep26813

**Published:** 2016-06-02

**Authors:** Cong Li, Wentao Cai, Chenghao Zhou, Hongwei Yin, Ziqi Zhang, Juan J. Loor, Dongxiao Sun, Qin Zhang, Jianfeng Liu, Shengli Zhang

**Affiliations:** 1 College of Animal Science and Technology, Key Laboratory of Animal Genetics and Breeding of Ministry of Agriculture, National Engineering Laboratory for Animal Breeding, China Agricultural University, Beijing 100193, China; 2 Department of Animal Sciences and Division of Nutritional Sciences, University of Illinois, Urbana, IL, 61801, USA

## Abstract

Paired-end RNA sequencing (RNA-Seq) was used to explore the bovine transcriptome from the mammary tissue of 12 Chinese Holstein cows with 6 extremely high and 6 low phenotypic values for milk protein percentage. We defined the differentially expressed transcripts between the two comparison groups, extremely high and low milk protein percentage during the peak lactation (HP vs LP) and during the non-lactating period (HD vs LD), respectively. Within the differentially expressed genes (DEGs), we detected 157 at peak lactation and 497 in the non-lactating period with a highly significant correlation with milk protein concentration. Integrated interpretation of differential gene expression indicated that *SERPINA1, CLU, CNTFR, ERBB2, NEDD4L, ANG, GALE, HSPA8, LPAR6* and *CD14* are the most promising candidate genes affecting milk protein concentration. Similarly, *LTF, FCGR3A, MEGF10, RRM2* and *UBE2C* are the most promising candidates that in the non-lactating period could help the mammary tissue prevent issues with inflammation and udder disorders. Putative genes will be valuable resources for designing better breeding strategies to optimize the content of milk protein and also to provide new insights into regulation of lactogenesis.

Milk production and composition are two of the most important economic traits for dairy cattle. An increase in the efficiency of milk protein synthesis is a highly desirable goal for the dairy industry, which also is an ongoing challenge^1^. With the generation and development of molecular quantitative genetics, identifying genes underlying milk protein traits and incorporating them into genetic evaluation systems would be valuable for dairy cattle breeding programs. In the past several decades, QTL mapping, candidate gene analysis, and genome-wide association study (GWAS) have been the main strategies to identify causal genes or mutations for milk yield and composition in dairy cows^2^,^3^,^4^. As such, they have provided a foundation for the generation of new biomarkers for trait selection. A large number of promising genomic regions and genetic associations have been identified, e.g. two confirmed causal mutations, DGAT1p.Lys232Ala and GHRp.Phe279Tyr^5^,^6^. Although these techniques have contributed significantly to our understanding of mechanisms on milk yield, component synthesis and metabolism, several major limitations still exist. A major one is being the inability to detect structural genomic aberrations and base mutations, which is the commonly existing challenge to identify the causal mutations.

The rapid development of next generation sequencing (NGS) technologies has overcome many of these problems^7^. NGS permits the investigation of an entire individual genome and transcriptome with unprecedented resolution and throughput^8^. Of these, RNA-Seq has been widely used to detect differentially expressed genes (DEGs) between two gene expression patterns, alternative splicing (AS) events, single nucleotide polymorphisms (SNPs) and insertion or deletion (InDels)^9^. In addition, RNA-seq promises to unravel previously inaccessible complexities in the transcriptome, such as allele-specific expression and novel promoters and isoforms^10^.

In bovine, many studies utilizing RNA-Seq have been conducted using adipose^11^, milk^12^, mammary tissue^13^, muscle^14^, liver^15^, embryo^16^, and immune and health traits^17^. Of these, limited studies on transcriptome of bovine mammary tissues have been reported. The identification and characterization of genes expressed in mammary tissue represents an important step toward understanding the complex biological properties of the mammary gland.

Herein, we report for the first time a complete dataset detailing the mammary tissue transcriptome from Chinese Holstein cows with extremely high or low milk protein percentage at peak lactation and also during the non-lactating period using RNA-Seq. To detect the effect of milk protein synthesis capacity, we compared the expression profiles of mammary tissues with high and low milk protein percentage cows; to test whether there was an effect of lactation stage on the expression of individual genes, the common DEGs of mammary tissues at peak and non-lactation periods were compared. We then conduct integrated analysis to propose key candidate genes affecting milk protein. Putative candidate genes identified could lead to improved selection of dairy cattle while providing new insights into milk protein traits.

## Results

### RNA sequencing of bovine mammary tissue

We acquired a total of 913.31 million clean reads with an average of 76.10 million (range, 66.40 to 88.01 million) for each sample. The quality value of Q20 and Q30 for sequencing was 96.40% and 89.59%, respectively (see Supplementary Table S1). Approximately 89.66% of the total reads uniquely mapped to the UMD 3.1 reference genome http://www.ncbi.nlm.nih.gov/genome/guide/cow/index.html. A total of 2.29% of sequences matched multiple positions in the reference genome, and 8.05% did not map to the reference genome (see Supplementary Table S2). Only the uniquely mapped reads were considered in this analysis.

### Gene expression level

Gene expression intensity was calculated using RPKM method and the results of all 12 samples are presented in Supplementary Table S3. We identified an average of 19,866 expressed genes (from 18,385 to 21,101) per sample among a total of 27,544 annotated bovine genes during the peak and non-lactating periods (see Supplementary Table S3). To better categorize these genes, which had differential expression levels, the gene expression RPKM values were categorized into five groups: high (≥60 RPKM), medium-to-high (15 to 60 RPKM), medium (3 to 15 RPKM), low-to-medium (1 to 3 RPKM) and low (≤1RPKM) (see Supplementary Table S3).

### Top genes expressed in the mammary tissue during the peak and non-lactating periods

The top 20 genes expressed in the mammary tissue at the peak lactation stage are shown in Table 1. Strikingly, the milk protein genes *CSN2, CSN1S1, LGB, CSN3, CSN1S2* and *LALBA* accounted for 71.33% of the mammary tissue total mRNA at peak lactation (see Supplementary Fig. S1). Compared with peak lactation, the mammary tissue transcriptome during the non-lactating period had a completely different rank of genes in terms of abundance, e.g. casein and whey protein genes were not highly expressed during the non-lactating period, but *COX1, EEF1A1, LTF, COX3, IGLL1, CD74* and *MT-CYB* were the top expressed genes. The top 20 genes expressed in the mammary tissue during the non-lactating period are presented in Table 2.

### Differentially expressed genes (DEGs)

**Figure 1:**
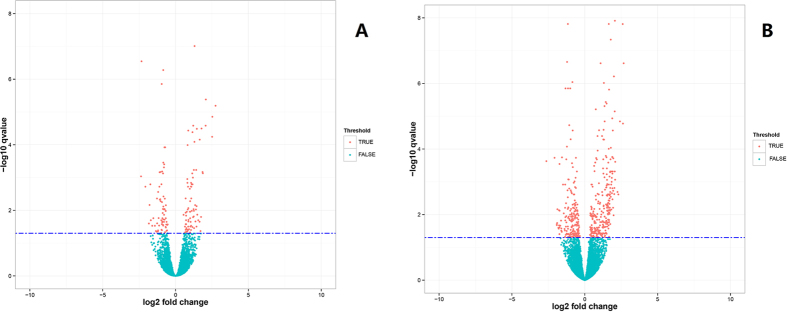
Volcano plot displaying differential expressed genes in bovine mammary tissues within two different comparison groups. (**A**) DEGs identified in bovine mammary tissues with high and low milk protein percentage at peak lactation. (**B**) DEGs identified in bovine mammary tissues with high and low milk protein percentage during the non-lactating period. The y-axis corresponds to the mean expression value of log_10_ (q-value), and the x-axis displays the log2 fold change value. The red dots represent the significantly differential expressed transcripts (q < 0.05); the blue dots represent the transcripts whose expression levels did not reach statistical significance (q > 0.05).

A total of 157 and 497 DEGs were detected in the high versus low milk protein percentage at peak lactation (HP vs LP), high versus low milk protein percentage during the non-lactating period (HD vs LD), respectively. Of these, 138 are known and 19 are novel in 157 genes, 450 are known and 47 are novel in 497 genes. In addition, *GALE, INSR, SERPINA1, IGFBP3, BMP1, SERPINA5, TMX1, MERTK* and *SLITRK6* were the only significant DEGs found for the high versus low milk protein percentage at peak lactation and the non-lactating period. The details of all DEGs in the two different comparison groups are shown in Supplementary Tables S4 and 5. Volcano plots of genes that are differentially expressed in the two comparison groups illustrate distinct transcriptional profiles (Fig. 1A,B).

**Figure 2:**
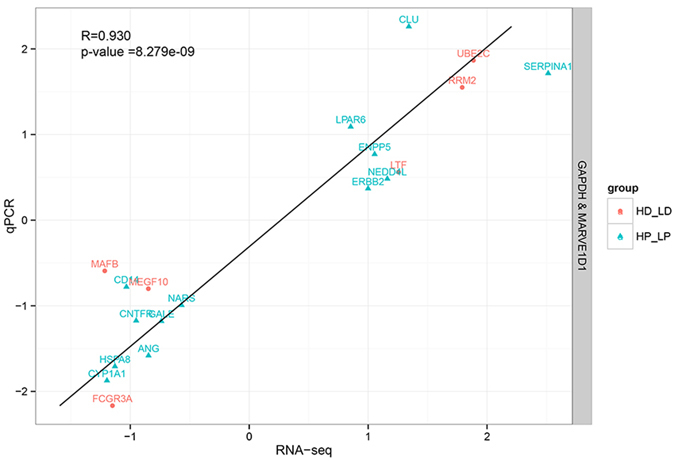
Correlations of mRNA expression level of 19 randomly differentially expressed genes in bovine mammary tissues between high and low milk protein percentage under peak and non-lactating period using RNA-Seq and qRT-PCR. The x- and y-axis show the log2 (ratio of mRNA levels) measured by RNA-seq and qRT-PCR, respectively. The DEGs marked with blue dots were detected between high and low milk protein percentage at peak lactation (HP vs LP), and the DEGs marked with red dots were identified between high and low milk protein percentage during non-lactating period (HD vs LD).

To validate the RNA-Seq results, 19 random DEGs including *ANG, CD14, CLU, CNTFR, CYP1A1, ENPP5, ERBB2, GALE, HSPA8, LPAR6, NARS, NEDD4L, SERPINA1, FCGR3A, LTF, MAFB, MEGF10, RRM2* and *UBE2C* were selected for qRT-PCR analysis. The comparisons of expression abundance of these 19 genes from qRT-PCR normalized to *MARVELD1, GAPDH* and RNA-Seq were showed in Supplementary Fig. S2. The correlations between the mRNA expression level from qRT-PCR and RNA-Seq were relatively high, with Pearson correlation coefficients of 0.86 (*P* = 8.28E-09) (Fig. 2), validating the repeatability and reproducibility of gene expression data in this study.

### GO and pathway analysis of the DEGs

Multiple pathways and GO terms including biological process, cellular component and molecular function were significantly enriched for these DEGs. The details of the significant pathways in the two comparison groups are presented in Supplementary Tables S6 and 7 and the significant GO terms are shown in Supplementary Tables S8 and 9. For milk protein traits, the important pathways identified were ‘Aminoacyl-tRNA biosynthesis’, ‘Cysteine and methionine metabolism’, ‘ECM-receptor interaction’ and ‘p53 signaling pathway’, which also involved several target candidate genes. Multiple significant GO terms are related to synthesis, transport, and metabolic process of AA and related proteins as well as insulin-like growth factor receptor signaling pathway. The top ten significantly enriched molecular functions for milk protein during peak lactation were associated with insulin-like growth factor binding, protein binding and transmembrane receptor protein kinase activity. The insulin-like growth factor receptor signaling pathway and lipid particles are the most significantly enriched biological processes and cellular components for milk protein during peak lactation, respectively. The six most significantly enriched molecular functions for milk protein during the non-lactating period were concentrated on peptidase regulator or inhibitor activity and DNA binding.

### Candidate genes

Combining the significant level and expression level of DEGs, GO and pathway results, QTL databases and gene function, allows us to suggest *SERPINA1, CLU, CNTFR, ERBB2, NEDD4L, ANG, GALE, HSPA8, LPAR6* and *CD14* as the 10 novel and promising candidate genes for milk protein synthesis, transport and metabolism during the peak lactation stage. In addition, 10 known genes (*WAP, NARS, MARS, GARS, CDO1, GATM, INSR, IGF1R, IGFBP3* and *CRIM1*) affecting milk protein traits also were revealed. The details of the above candidate genes identified in the comparison HP versus LP are listed in Table 3. For the comparison of HD versus LD, *SERPINA1, GALE, INSR* and *IGFBP3* were identified as candidates for milk protein, while the majority of genes were associated with immune responses and diseases, of these, *LTF, FCGR3A, MEGF10, RRM2* and *UBE2C* can be considered as novel promising candidates for counteracting stress, inflammation and disease (Table 4).

## Discussion

In this study, we obtained a comprehensive landscape of genes associated with milk protein in the context of transcriptome profiles across 12 mammary tissue samples during two different stages of lactation. Importantly, the use of longitudinal biopsies of mammary tissue allowed for a direct and comprehensive landscape of the transcriptome in the intact organ. Our findings provide novel and valuable insights for lactogenesis as well as yield a suite of molecular breeding resources to optimize the content of milk proteins.

The number of DEGs in the high milk protein versus the low milk protein, and between the peak lactation versus non-lactating period was considerably different. A total of 157 genes were found to differ significantly in expressional level between HP and LP, while some of the genes with a known function, e.g. *DGAT1*^5^, *GHR*^6^, S*CD*^18^, for milk production and composition did not differ. It is likely that these genes with great effects have been fixed through long-term genetic selection, thus, no large differences are observed between the high and low milk protein percentage groups. In addition, the six milk protein genes, *CSN2, CSN1S1, LGB, CSN3, CSN1S2* and *LALBA* also did not reach significance. In spite of that, we still consider these milk protein genes as the main reason underlying the different phenotype between the milk protein percentage groups. The lack of effect is probably due to the inability of the RNA-Seq software to identify differential expression in transcripts because of the large number of reads of the caseins, genetic polymorphisms of the target genes that affect milk protein composition, and the regulation and interaction of other minor DEGs with the six major protein coding genes. Therefore, based on DEGs results from RNA-Seq combined with the statistical significance and expression level of DEGs plus QTLs information and all the bioinformatics analyses we were able to identify *SERPINA1, CLU, CNTFR, ERBB2, NEDD4L, ANG, GALE, HSPA8, LPAR6* and *CD14* as novel promising candidate genes underlying milk protein synthesis, transport and metabolism.

Serpin Peptidase Inhibitor, Clade A (Alpha-1 Antiproteinase, Antitrypsin), Member 1 (*SERPINA1*), is a member of the serine protease inhibitor (serpin) superfamily of proteins, which inhibits a wide variety of proteases including trypsin, chymotrypsin, thrombin, kallikrein and elastase^19^. In our study, the expression abundance in HD vs LD (4,028 reads) was nine-fold higher than in HP vs LP (492 reads), revealing its high expression and main function in the non-lactating period. *SERPINA1* is present in relatively high concentration in human milk as well as in bovine and porcine colostrum^20^. It is likely that the high expression of *SERPINA1* started from the end of the lactation or non-lactating period extending through the colostrum period. Milk protease inhibitors influence both mother and infant development, probably through inactivating some endogenous proteases, affecting local proteolytic activity within the mammary tissue during colostrum formation, or increasing the survival of other milk proteins^19^. *SERPINA1* levels are reported to affect milk composition and quality^21^. In addition, associations of polymorphisms of the *SERPINA1* gene with milk production traits in dairy cattle were demonstrated^22^,^23^,^24^. Cows with the GCGGC *SERPINA1* haplotype had a superior genetic merit for milk protein yield^22^. Clusterin (*CLU*), a widely expressed glycoprotein, is induced during apoptosis and stress in hormone-dependent tissues including the mammary gland^25^. Clusterin has been proposed to be a secreted mammalian chaperone^26^. The effect of *CLU* on milk production traits in Chinese Holstein cows was reported previously^27^.

Ciliary neurotrophic factor (*CNTF*) exerts its biological functions through its receptor *CNTFR* to activate multiple downstream signaling pathways, such as AMPK, Jak2-Stat5, MAPK and PI3K-AKT^28^. As is known, these networks have critical roles in milk protein synthesis regulation^29^,^30^. In addition, CNTF induced the dephosphorylation of a set of proteins and phosphorylation of a different set^31^. It is suggested that *CNTFR* might be a novel promising gene for milk protein synthesis through these known networks. Erb-b2 receptor tyrosine kinase 2 (*ERBB2*), encodes a member of the epidermal growth factor (EGF) receptor family of receptor tyrosine kinases^32^. The mammary gland of transgenic mice that overexpressed dominant negative versions of *ERBB2* also contained regions in which alveolar clusters more typical of late pregnancy were present during lactation, the influence of an *ERBB2* transgene on lactogenesis was estimated by its influence on milk protein gene expression^33^. In addition, heterodimers of *ERBB2* and *ERBB3* activate PI3K signaling by direct binding of PI3K regulatory subunit p85 to phosphorylated tyrosine residues, which is known to regulate milk protein synthesis^29^.

Neural precursor cell expressed, developmentally down-regulated 4-like, E3 ubiquitin protein ligase (*NEDD4L*), encodes a ubiquitin ligase that targets the epithelial sodium channel for degradation. *NEDD4L* plays a broader role as a general modulator of Smad turnover during TGF-β signal transduction^34^. TGF-β is a member of a family of growth factors that have been shown to affect the maturation and function of normal mammary gland^35^. For example, overexpression of TGF-β in the mammary glands of transgenic mice decreased milk protein production^36^. In mammary tissue explants from mid-pregnant mice, TGF-β can inhibit β-casein production by a post-transcriptional mechanism^37^. In addition, TGF-β can induce expression of extracellular matrix (ECM) proteins by human mammary epithelial cells in culture^38^. Considering the effects on mammary gland patterning, *NEDD4L* appears to play a role in regulating accumulation of milk proteins during lactation via TGF-β. Bovine angiogenin (*ANG*) is a constituent of milk that is mainly responsible for the effect of milk consumption in suppressing bone resorption^39^. An important role of *ANG* during lactation in bovine mammary tissue is associated with a continuous formation of blood vessels. The blood flow across the mammary tissue increases dramatically at the onset of lactation^40^. A positive correlation between blood flow and milk yield has been demonstrated^40^. Up-regulating *ANG* may be associated with the promotion of PI3K/Akt/mTOR signaling pathway^41^, regulating milk protein synthesis^29^,^30^.

UDP-galactose-4-epimerase (*GALE*), encodes UDP-galactose-4-epimerase which catalyzes two distinct but analogous reactions: the epimerization of UDP-glucose to UDP-galactose, and the epimerization of UDP-N-acetylglucosamine to UDP-N-acetylgalactosamine. String interaction network showed that GALE protein interacts with lactalbumin, alpha (*LALBA*), UDP-Gal: betaGlcNAc beta 1,4- galactosyltransferase, polypeptide 1 (*B4GALT1*) and UDP-glucose 6-dehydrogenase (*UGDH*). Of these, *LALBA* is known as major milk protein and is a subunit of lactose synthase. As one of the best studied glycosyltransferases, *B4GALT1* is responsible for the synthesis of complex-type N-liked oligosaccharides in many glycoproteins^42^. In addition, an association of polymorphisms of the *B4GALT1* with milk production traits in Holstein cows has been reported^43^. The *UGDH* gene was shown to be associated with milk yield and milk composition^44^. It is suggested that *GALE* functions through interacting with known genes for milk production and composition.

Heat shock 70kDa protein 8 (*HSPA8*) functions as an ATP-dependent molecular chaperone that facilitates folding of newly synthesized polypeptides, assembly of multiprotein complexes, transport of proteins across cellular membranes, and lysosomal degradation of proteins^45^. In addition, *HSPA8* is an important gene in the proposed network of milk protein synthesis regulation encompassing MAPK^30^. The p38 MAPK has a positive effect on protein synthesis by increasing the stability of mRNA through phosphorylation of the AU-rich element-binding protein^46^. A putative role for *HSPA8* influencing milk protein synthesis was suggested using a proteomic approach^47^.

As the receptor for lysophosphatidic acid (LPA), lysophosphatidic acid receptor 6 (*LPAR6/P2RY5*) encodes an orphan G protein-coupled receptor. LPA stimulated the growth of normal mammary epithelial cells from mature virgin mice^48^. *LPAR6* is involved in the ‘PI3K-Akt signaling pathway’, which may function as an LPA receptor in the milk protein synthesis. As an immune gene, *CD14* is a pattern recognition receptor for bacterial lipopolysaccharide (LPS). Immunoprecipitation of *CD14* from milk and *in vitro* digests demonstrated that *CD14* is able to complex with other milk proteins, namely, α-lactalbumin, which protects it from degradation^49^. In addition, *CD14* was included in the LXR/RXR activation pathway associated with milk protein^50^. The G allele of *CD14*-1908 had an association with lower milk fat and protein yields^23^.

In addition to above genes, 10 known genes (*WAP, NARS, MARS, GARS, GATM, CDO1, INSR, IGF1R, IGFBP3* and *CRIM1*) affecting milk protein traits were also revealed. Whey acidic protein (*WAP*) is a kind of whey proteins, which together with caseins are the main proteins in milk^30^. Aminoacyl-tRNA synthetases are enzymes involved in protein biosynthesis catalyzing the specific attachment of AA to their cognate tRNAs^51^. As the family of tRNA synthetases, asparaginyl-tRNA synthetase (*NARS*), methionyl-tRNA synthetase (*MARS*) and glycyl-tRNA synthetase (*GARS*) may be core signal mediators in addition to their catalytic roles in milk protein synthesis. Glycine amidinotransferase (*GATM*) and cysteine dioxygenase type 1 (*CDO1*) are understood to be one of the key enzymes in the specific AA biosynthesis, respectively^52^,^53^. The insulin receptor (*INSR*), insulin-like growth factor 1 receptor (*IGF1R*) and insulin-like growth factor binding protein 3 (*IGFBP3*) are involved in the insulin pathway, which is known to regulate milk protein synthesis^54^. Cysteine rich transmembrane BMP regulator 1 (*CRIM1*), a novel gene encodes a cysteine-rich repeat protein containing an IGF-binding protein motif and an insulin-like growth factor binding protein motif^55^.

Most of the DEGs in HD vs LD were associated with immune response, inflammation and disease, i.e. *LTF, FCGR3A, RRM2, UBE2C* and *MEGF10.* This result underscores the importance of “prevention” within the mammary tissue during the non-lactating period. Nevertheless, the above results indicated that bovine mammary tissue relies heavily on transcriptional regulation of genes to induce copious milk synthesis and secretion, which confirms the original work by Bionaz *et al*.^1^.

Deriving gene networks and pathways is an effective strategy to elucidate the mechanisms underlying the genetic variability of milk protein traits. Different genes usually cooperate with each other to exercise their biological functions and pathway-based analysis helps to further understand the biological functions of genes^11^. For DEGs identified in mammary glands with different milk protein percentage during peak lactation, the significant pathways and GO terms are mainly associated with biosynthesis, transport, metabolism of AA and proteins. During the non-lactating period, significant GO terms and pathways containing significant DEGs for milk protein are intensively enriched for inflammation response, disease and immune-related function. The results agree with previous gene expression studies conducted in the mouse mammary tissue where immune related genes showed increased expression toward the later stages of lactation^56^.

The present data indicate that immune defense is a hallmark of the non-lactating period, massive development of the protein synthesis infrastructure and promotion of protein transportation is a hallmark of the peak lactation stage. Thus, the immune response in mammary tissue is pivotal due to the need for preventing pathogen-causing mastitis^57^. The number of DEGs in HD versus LD (497 DEGs) was approximately three times higher than HP versus LP (157 DEGs), indicating a larger fluctuation of milk protein percentage during the non-lactating period. A higher expression level was observed during the peak lactation (196,875,675 reads) than the non-lactating period (149,931,847 reads), including six milk protein genes. This result is similar to data from mammary gland of sheep^58^ and human^59^. Based on available publicly microarray datasets, patterns of transcription of six milk protein genes showing the highest expression was observed at peak lactation in dairy cows^1^. It is reasonable to expect that lactation requires the increase in expression of a greater number of genes. However, lower expression level with a wider variety of genes were expressed during the non-lactating period (23,302 genes) compared with peak lactation (21,403 genes). This suggests that greater numbers of genes start to initiate expression in the non-lactating period to prepare well for parturition, partly to synthesize colostrum for the calf.

The majority of genes supporting lactation are already expressed at a high level at the late pregnant stage^1^, which was confirmed by our findings. Thus, such changes of physiological function are inevitably regulated by a series of related spatio-temporal gene expression and signaling pathways. Research on human milk revealed that during the transition to lactation there was the lowest complexity in the transcriptome with a smaller number of genes contributing to a larger fraction of the total mRNA while peak lactation milk had the highest complexity^59^. However, in our study, peak lactation mammary tissue had lower complexity in the transcriptome with a smaller number of genes contributing to a larger fraction of the total mRNA while non-lactating mammary tissue had a higher complexity. Such differences are probably due to the milk secretion reaching a plateau level and the six milk protein genes accounting for 71% of the total expressed mRNA at peak lactation while the remaining 29% of expressed genes only playing limited role in the process. The deduction was also confirmed by the distribution of genes expressed across intervals that the larger number of medium-to-higher expressed genes in non-lactating period compared to peak lactation, the smaller number of low expressed genes in non-lactating period than peak lactation.

The number of DEGs for different milk protein percentages at peak lactation (157) and the non-lactating period (497) were vastly different, and only 9 common genes were detected for the two comparison groups. This response indicated that stage of lactation has great influence on milk protein traits. Therefore, when exploring the candidate genes contributing to the extremely different phenotypes for milk protein it is necessary to consider the differences in stage of lactation. The overall analysis indicated that the bovine mammary tissue relies heavily on a coordinated transcriptional regulation to begin and end lactation.

## Conclusions

This is the first study to apply the recently developed NGS technology to analyze the expression profiles of bovine mammary tissues with different milk protein percentages at different stages of lactation. Approximate 20,000 of 27,544 genes annotated in NCBI UMD3.1 bovine genome assembly were ubiquitously expressed in mammary tissues. The highest expression level was observed in peak lactation, especially for *CSN2, CSN1S1, LGB, CSN3, CSN1S2* and *LALBA*, making up 71% of the total pool of mRNA in this stage of lactation.

*SERPINA1, CLU, CNTFR, ERBB2, NEDD4L, ANG, GALE, HSPA8, LPAR6* and *CD14* can be used as novel promising candidate genes for milk protein synthesis and metabolism, *LTF, FCGR3A, MEGF10, RRM2* and *UBE2C* as novel promising candidates for counteracting stress, inflammation and diseases to prepare well for parturition. Our findings will facilitate the understanding of the milk protein molecular synthesis and milk secretion, and provide compacted sound basis for designing further studies on the function of candidate genes through protein and cellular levels.

## Materials and Methods

### Ethics statement

All procedures for animal handling prior to and after mammary gland biopsy were conducted under protocols approved by the Animal Welfare Committee of China Agricultural University (Permit Number: DK996). And all experiments were performed in accordance with approved relevant guidelines and regulations.

### Animals and experimental design

Twelve multiparous and healthy mastitis-free Chinese Holstein cows at the Beijing Sanyuan Dairy Farm Center were selected for the study. Routine standard performance tests, i.e. Dairy Herd Improvement system (DHI) have been carried out since 1999. The average milk protein percentage in this population was 3.1% (2.7~3.8%). Based on the DHI data, we defined a high milk protein percentage group as those cows with 3.5% protein and the low milk protein percentage group was composed of cows with 3.0% protein throughout previous lactation. We constructed two comparison groups: high milk protein percentage and low milk protein percentage at peak lactation (HP vs LP) and non-lactating period (HD vs LD). Three cows were sampled in each group. The cows were kept in a free stall housing, fed a total mixed ration (TMR) and had access to water ad libitum. Cows were milked three times daily in the milking parlor. A total of 6 biopsy samples were collected at approximately 60 days postpartum (peak lactation), and the other 6 samples during the non-lactating period (~30 days after dry-off).

Mammary tissue biopsies were taken 1~3 h after milking. The biopsy procedure was performed according to the method of Schmitz *et al*. with modifications^60^. Briefly, the skin of the selected biopsy site was first shaved and disinfected with ethanol (75%), then anaesthetized with SU-MIAN-XIN (846 compound anesthetic agent, 30 to 40 mg, intravenously) (China Agricultural University Veterinary Teaching Hospital), and injected subcutaneously with 1 mL of procaine (China Agricultural University Veterinary Teaching Hospital). A 1.5 cm incision was made in the skin at the midpoint of a rear quarter of the mammary gland and connective tissue using shears and tweezers was blunt-dissected away exposing the secretory gland capsule. The mammary tissues biopsy (~500 mg) was then obtained and immediately frozen in liquid nitrogen and stored at −80 °C until RNA isolation. The suture was tied as the cannula was removed and pressure applied to reduce collection of blood under the skin. Immediately after the experiment, all 12 cows received antibiotic prophylaxis and anti-inflammatory therapy.

### RNA extraction

Total RNA was extracted from the bovine mammary tissue via the Trizol method (Invitrogen, Carlsbad, CA) according to the manufacturer’s instructions. RNA degradation and contamination was monitored on 1% agarose gels, the purity and concentration was measured using the NanoPhotometer^®^ spectrophotometer (IMPLEN, CA, USA) and Qubit^®^ RNA Assay Kit in Qubit^®^ 2.0 Flurometer (Life Technologies, CA, USA), respectively. RNA integrity was assessed with the RNA Nano 6000 Assay Kit of the Bioanalyzer 2100 system (Agilent Technologies, CA, USA). The 12 purified RNA samples had a RIN ≥ 7.0 and the yielded ≥5.0 total μg RNA. They were used for paired-end sequencing.

### Library preparation and RNA sequencing

A total of 3 μg RNA per sample was used as input material. Sequencing libraries were constructed using NEBNext^®^ Ultra™ RNA Library Prep Kit for Illumina^®^ (NEB, USA) following manufacturer’s recommendations and index codes were added to attribute sequences to each sample. The index-coded samples were clustered on a cBot Cluster Generation System using TruSeq PE Cluster Kit v3-cBot-HS (Illumia) according to the manufacturer’s instructions. After cluster generation, the library preparations were sequenced on an Illumina Hiseq 2000 platform and 100 bp paired-end reads were generated. The sequenced RNA-Seq raw data for 12 bovine mammary tissues is available from NCBI Sequences Read Archive with accession number SRP065563 and SRP065827.

### Quality control for paired-end reads

Raw data (raw reads) of fastq format were first processed using in-house perl scripts. In this step, clean data (clean reads) were obtained by removing reads containing adapters, reads containing ploy-N and low quality reads from raw data. At the same time, Q20 (the proportion of bases with a phred base quality score greater than 20, i.e., the proportion of read bases whose error rate is less than 1%), Q30 (the proportion of bases with a phred base quality score greater than 30, i.e., the proportion of read bases whose error rate is less than 0.1%) and GC content of the clean data were calculated. All the downstream analyses were based on the clean data.

### Reads mapping on the bovine reference genome and gene expression analysis

The bovine genome UMD3.1 ( ftp://ftp.ensembl.org/pub/release-79/fasta/bos_taurus/dna/) was utilized as the reference genome for the assembly. Index of the reference genome was built using Bowtie v2.0.6^61^ and paired-end clean reads were aligned to the reference genome using TopHat v2.0.9^9^,^62^ ( http://tophat.cbcb.umd.edu/). Also, a database of splice junctions were generated by TopHat based on the gene model annotation files^62^,^63^ ( ftp://ftp.ensembl.org/pub/release-77/gtf/bos_taurus).

HTSeq v0.6.1 was used to count the reads numbers mapped to each gene^64^. Transcript abundances were estimated as reads per kilobase of exon model per million mapped reads (RPKM)^8^, which was calculated based on the length of the gene and reads count mapped to this gene. A gene was defined as expressed if it was detected above 0.01 RPKM in any given sample^8^.

### Differential expression analysis

The DEGs and transcript analysis across Holstein cows with high and low milk protein percentage during peak and non-lactating periods (i.e., HP vs LP, HD vs LD) were performed using the DESeq2 R package (1.8.1)^65^. DESeq2 provides statistical routines for determining differential expression in digital gene expression data using a generalized linear model based on the negative binomial distribution, the estimates of dispersion and logarithmic fold changes incorporate data-driven prior distributions^65^. The DESeq2 package performs independent filtering. RNA-Seq read counts were modeled by a generalized linear model considering the experimental design, with two phenotypes (high milk protein percentage and low milk protein percentage) and two stages of lactation (peak lactation and non-lactating period). The model for the HP vs LP and HD vs LD comparisons only included the phenotype factor. The statistical power of this experimental design was estimated by a power analysis tool ( http://www2.hawaii.edu/~lgarmire/RNASeqPowerCalculator.htm) with one-factor design model^66^, which reached above 0.95 (Supplementary Fig. S3). The resulting *P*-values were adjusted using Benjamini and Hochberg’s approach for controlling the false discovery rate. The fold changes (in log_2_ scale), *p*-values and *q*-values (false discovery rate corrected *p* values) of the DEGs were reported in the output files from DESeq2. Genes with a *q*-value < 0.05 were assigned as differentially expressed.

### Gene ontology (GO) and pathway enrichment analysis of DEGs

GO and pathway enrichment analysis of DEGs was implemented in the GOstats R package (2.34.0)^67^, in which gene length bias was corrected. GO terms and KEGG pathways ( http://www.genome.jp/kegg/) with *P*-value less than 0.05 were considered significantly enriched by DEGs.

### Confirmation of RNA-Seq results with qRT-PCR

To confirm the sequencing results, qRT-PCR was performed on 19 randomly selected DEGs. Total RNA was reverse-transcribed to cDNA using PrimeScript RT reagent Kit with gDNA Eraser (TaKaRa) according to the manufacturer’s instructions. Primers were designed via Primer Express 3.0.1 software (Applied Biosystems) and are shown in Supplementary Table S10. QRT-PCR was carried out in triplicate with the LightCycler^®^ 480 SYBR Green I Master Kit (Roche) in a 15 μL reaction on a LightCycle480 (Roche Applied Science, Penzberg, Germany), using the following program: 95 °C for 10 min, 45 cycles of 95 °C for 10s, 60 °C for 10s, and 72 °C for 10s, 72 °C for 6 min. The relative gene expression values were calculated using the 2^−ΔΔCt^ method. The mRNA levels of the DEGs were normalized against two internal controls, *MARVELD1* and *GAPDH*, in all 12 mammary tissue samples. These two genes are commonly used as control genes^68^ and were stably expressed in mammary tissues in this study. Finally, the correlations between the mRNA expression level from qRT-PCR and RNA-Seq for 19 genes were estimated using R (V3.2).

## Additional Information

**How to cite this article**: Li, C. *et al*. RNA-Seq reveals 10 novel promising candidate genes affecting milk protein concentration in the Chinese Holstein population. *Sci. Rep.* **6**, 26813; doi: 10.1038/srep26813 (2016).

## Supplementary Material

Supplementary Information

Supplementary Dataset 1

Supplementary Dataset 2

Supplementary Dataset 3

Supplementary Dataset 4

Supplementary Dataset 5

Supplementary Dataset 6

## Figures and Tables

**Table 1:** Top 20 expressed genes in the mammary tissues at peak lactation.

**Gene name**	**No. reads**	**Gene description**	**Gene function**
*CSN2*	52798009	Casein beta	Major milk protein, important role in determination of the surface properties of the casein micelles, primary source of essential amino acids
*CSN1S1*	41418928	Casein alpha s1	Major milk protein, important role in the capacity of milk to transport calcium phosphate
*LGB*	13940973	Beta-lactoglobulin	Major milk protein, form a complex with fatty acids, a dominant allergen in cow’s milk
*CSN3*	12996425	Casein kappa	Major milk protein, stabilizes micelle formation, preventing casein precipitation in milk, primary source of essential amino acids
*CSN1S2*	12986457	Casein alpha-S2	Major milk protein, important role in the capacity of milk to transport calcium phosphate
*LALBA*	6299787	Lactalbumin, alpha	Major milk protein, regulatory subunit of lactose synthase, a mammary epithelial-specific protein
*GLYCAM1*	2543039	Glycosylation-dependent cell adhesion molecule 1	Pseudogene
*COX1*	1962773	Cytochrome c oxidase subunit I	Catalytic subunit of the enzyme, catalyzes the reduction of oxygen to water
*FASN*	752434	Fatty acid synthase	Catalyzes the formation of long-chain fatty acids from acetyl-CoA, alonyl-CoA and NADPH
*COX3*	620201	Cytochrome c oxidase subunit III	Subunits I, II and III form the functional core of the enzyme complex
*MT-CYB*	563159	Mitochondrially encoded cytochrome B	Component of the biquinol-cytochrome c reductase complex
*XDH*	486431	Xanthine dehydrogenase	Oxidative metabolism of purines, essential for envelopment of milk fat globules
*MFGE8*	472494	Milk fat globule-EGF factor 8 protein	Maintenance of intestinal epithelial homeostasis and the promotion of mucosal healing
*EEF1A1*	449681	Eukaryotic translation elongation factor 1 alpha 1	Subunit of elongation factor-1 complex, translation of proteins
*GPAM*	376487	Glycerol-3-phosphate acyltransferase, mitochondrial	Encodes a mitochondrial enzyme, involving in glycerolipid biosynthesis
*ATP6*	346573	ATP synthase F0 subunit 6	Hydrogen ion transmembrane transporter activity
*MT-ND3*	344480	Mitochondrially encoded NADH dehydrogenase 3	Core subunit of the mitochondrial membrane respiratory chain NADH dehydrogenase
*ND1*	323003	NADH dehydrogenase subunit 1	NADH dehydrogenase (ubiquinone) activity, oxidation-reduction process
*MT-ND4*	307483	Mitochondrially encoded NADH dehydrogenase subunit 4	NADH dehydrogenase (ubiquinone) activity, mitochondrial electron transport, NADH to ubiquinone
*SPP1*	301080	Secreted phosphoprotein 1	Milk protein, up-regulates interferon-gamma and IL-12

**Table 2:** Top 20 expressed genes in the mammary tissues during the non-lactating period.

**Gene name**	**No. reads**	**Gene description**	**Gene function**
*COX1*	2240820	Cytochrome c oxidase subunit I	Catalytic subunit of the enzyme, catalyzes the reduction of oxygen to water
*EEF1A1*	1587546	Eukaryotic translation elongation factor 1 alpha 1	Subunit of elongation factor-1 complex, translation of proteins
*LTF*	1254103	Lactotransferrin	Milk protein, iron binding protein, bactericidal and antiviral functions
*COX3*	803125	Cytochrome c oxidase subunit III	Subunits I, II and III form the functional core of the enzyme complex
*IGLL1*	682619	Immunoglobulin lambda-like polypeptide 1	Critical for B-cell development
*CD74*	676236	CD74 molecule, major histocompatibility complex, class II invariant chain	Important role in MHC class II antigen processing by stabilizing peptide-free class II alpha/beta heterodimers in a complex
*MT-CYB*	645897	Mitochondrially encoded cytochrome b	Component of the ubiquinol-cytochrome c reductase complex
*PABPC1*	564070	Poly(A) binding protein, cytoplasmic 1	Binds the poly(A) tail of mRNA, involved in cytoplasmic regulatory processes of mRNA metabolism
*B2M*	552968	Beta-2-microglobulin	Component of the class I major histocompatibility complex (MHC), involved in the presentation of peptide antigens to the immune system
*COL3A1*	549800	Collagen, type III, alpha 1	Involved in regulation of cortical development
*TPT1*	509911	Tumor protein, translationally-controlled 1	Protein binding, immune response
*SPP1*	485028	Secreted phosphoprotein 1	Milk protein, up-regulates interferon-gamma and IL-12
*RPS3A*	441003	Ribosomal protein S3A	Ribosomal protein, play a role during erythropoiesis through regulation of transcription factor DDIT3
*MT-ND4*	434700	Mitochondrially encoded NADH dehydrogenase subunit 4	NADH dehydrogenase (ubiquinone) activity, mitochondrial electron transport, NADH to ubiquinone
*MT-ND3*	419628	Mitochondrially encoded NADH dehydrogenase 3	Core subunit of the mitochondrial membrane respiratory chain NADH dehydrogenase
*CSN3*	416587	Casein kappa	Major milk protein, stabilizes micelle formation, preventing casein precipitation in milk, primary source of essential amino acids
*ND1*	411254	NADH dehydrogenase subunit 1	NADH dehydrogenase (ubiquinone) activity, oxidation-reduction process
*AHNAK*	408900	AHNAK nucleoprotein	Required for neuronal cell differentiation
*ATP6*	403220	ATP synthase F0 subunit 6	Hydrogen ion transmembrane transporter activity
*EEF2*	402263	Eukaryotic translation elongation factor 2	An essential factor for protein synthesis, catalyzes the GTP-dependent ribosomal translocation step during translation elongation

**Table 3:** Expression changes of the candidate genes in bovine mammary tissue with high milk protein percentage compared to low milk protein percentage at peak lactation.

**Symbol**	**CHR**	**No. Reads**	**Log2 fold change**	**Gene name**	**q-value**
*SERPINA1*	21	492.04	2.51	Serpin peptidase inhibitor, clade A, member 1	1.17E-09
*CLU*	8	2964.18	1.34	Clusterin	6.93E-03
*CNTFR*	8	991.80	−0.95	Ciliary neurotrophic factor receptor	1.40E-06
*ERBB2*	19	638.15	1.00	Erb-b2 receptor tyrosine kinase 2	5.25E-03
*NEDD4L*	24	796.05	1.16	Neural precursor cell expressed, developmentally down-regulated 4-like, E3 ubiquitin protein ligase	4.13E-05
*ANG*	10	4945.54	−0.85	Angiogenin, ribonuclease, RNase A family, 5	5.27E-07
*GALE*	2	609.70	−0.74	UDP-galactose-4-epimerase	4.41E-03
*HSPA8*	15	9488.17	−1.13	Heat shock 70kDa protein 8	2.91E-02
*LPAR6*	12	488.54	0.85	Lysophosphatidic acid receptor 6	1.90E-03
*CD14*	7	2619.05	−1.03	CD14 molecule	6.78E-04
*WAP*	19	40.75	−3.84	WAP four-disulfide core domain protein 18-like	1.62E-15
*NARS*	24	2664.23	−0.57	Asparaginyl-tRNA synthetase	2.78E-02
*MARS*	5	930.09	−0.85	Methionyl-tRNA synthetase	1.89E-02
*GARS*	4	1681.16	−0.90	Glycyl-tRNA synthetase	1.74E-02
*CDO1*	10	415.98	1.20	Cysteine dioxygenase type 1	1.61E-02
*GATM*	10	73.89	1.04	Glycine amidinotransferase	2.25E-02
*INSR*	7	556.70	0.69	Insulin receptor	4.33E-03
*IGF1R*	21	528.35	0.80	Insulin-like growth factor 1 receptor	1.11E-03
*IGFBP3*	4	680.00	0.81	Insulin-like growth factor binding protein 3	1.45E-03
*CRIM1*	11	661.78	0.82	Cysteine rich transmembrane BMP regulator 1	1.02E-04

**Table 4:** Expression changes of the candidate genes in bovine mammary tissue with high milk protein percentage compared to low milk protein percentage during the non-lactating period.

**Symbol**	**CHR**	**No. Reads**	**Log2** **fold change**	**Gene name**	**q-value**
*LTF*	22	207337.87	1.26	Lactotransferrin	2.52E-02
*FCGR3A*	3	735.60	−1.15	Fc fragment of IgG, low affinity IIIa, receptor (CD16a)	1.42E-06
*MEGF10*	7	1083.21	−0.85	Multiple EGF-like-domains 10	9.10E-07
*RRM2*	11	696.54	1.79	Ribonucleotide reductase M2	9.19E-05
*UBE2C*	13	270.35	1.89	Ubiquitin-conjugating enzyme E2C	1.16E-05
*SERPINA1*	21	4027.72	0.72	Serpin peptidase inhibitor, clade A, member 1	2.58E-04
*INSR*	7	2445.44	−0.80	Insulin receptor	3.92E-09
*GALE*	2	1223.02	0.91	UDP-galactose-4-epimerase	2.52E-02
*IGFBP3*	4	7944.35	−0.47	Insulin-like growth factor binding protein 3	3.59E-02
