# Supplementary material for: RNA-Seq reveals 10 novel promising candidate genes affecting milk protein concentration in the Chinese Holstein population
Source: Sci Rep. 2016 Jun 2;6:26813. doi: 10.1038/srep26813 (PMC4890585; doi:10.1038/srep26813)
Supplement: Supplementary Information [file srep26813-s1.doc]

**RNA-Seq reveals 10 novel promising candidate genes affecting milk protein concentration in the Chinese Holstein population**

Cong Li^1†^, Wentao Cai^1†^, Chenghao Zhou^1^, Hongwei Yin^1^, Ziqi Zhang^1^, Juan. J. Loor^2^, Dongxiao Sun^1^, Qin Zhang^1^, Jianfeng Liu^1^, Shengli Zhang^1^*****

**^1^**College of Animal Science and Technology, Key Laboratory of Animal Genetics and Breeding of Ministry of Agriculture, National Engineering Laboratory for Animal Breeding, China Agricultural University, Beijing 100193, China

^2^Department of Animal Sciences and Division of Nutritional Sciences, University of Illinois, Urbana, IL, 61801, USA

*****Corresponding author

Email: [zhangslcau@cau.edu.cn](mailto:zhangslcau@cau.edu.cn); zhang62733697@163.com

^†^These authors contributed equally to this work.

**Supplementary Table S1. The basic statistics for RNA-seq reads generated from mammary tissues of 12 cows with different milk protein percentage during the different milk lactation stage**

| Sample name^a^ | Raw reads | Clean reads | Clean bases | Error rate (%) | Q20^b^ (%) | Q30^c^ (%) | GC content (%) |
| --- | --- | --- | --- | --- | --- | --- | --- |
| HP_1_1 | 37341028 | 36077146 | 3.61G | 0.03 | 97.44 | 91.83 | 46.92 |
| HP_1_2 | 37341028 | 36077146 | 3.61G | 0.04 | 96.11 | 89.86 | 46.95 |
| HP_2_1 | 46149745 | 44008462 | 4.40G | 0.03 | 97.19 | 91.19 | 46.90 |
| HP_2_2 | 46149745 | 44008462 | 4.40G | 0.04 | 96.41 | 89.74 | 46.92 |
| HP_3_1 | 40874439 | 38724841 | 3.87G | 0.03 | 96.92 | 90.42 | 46.62 |
| HP_3_2 | 40874439 | 38724841 | 3.87G | 0.04 | 95.89 | 88.24 | 46.68 |
| LP_1_1 | 39877683 | 37792939 | 3.78G | 0.03 | 97.09 | 90.71 | 46.89 |
| LP_1_2 | 39877683 | 37792939 | 3.78G | 0.04 | 95.70 | 87.92 | 46.94 |
| LP_2_1 | 39307205 | 37997672 | 3.80G | 0.03 | 97.46 | 91.82 | 46.87 |
| LP_2_2 | 39307205 | 37997672 | 3.80G | 0.04 | 96.15 | 89.86 | 46.96 |
| LP_3_1 | 35908603 | 34678136 | 3.47G | 0.03 | 97.49 | 91.95 | 46.75 |
| LP_3_2 | 35908603 | 34678136 | 3.47G | 0.04 | 96.11 | 89.86 | 46.81 |
| HD_1_1 | 35598163 | 33955630 | 3.40G | 0.03 | 96.97 | 90.52 | 49.22 |
| HD_1_2 | 35598163 | 33955630 | 3.40G | 0.04 | 95.46 | 88.22 | 49.31 |
| HD_2_1 | 41696112 | 39168246 | 3.92G | 0.04 | 96.73 | 90.05 | 49.16 |
| HD_2_2 | 41696112 | 39168246 | 3.92G | 0.04 | 95.89 | 88.44 | 49.22 |
| HD_3_1 | 42125062 | 39582311 | 3.96G | 0.04 | 96.79 | 90.12 | 49.46 |
| HD_3_2 | 42125062 | 39582311 | 3.96G | 0.04 | 95.87 | 88.30 | 49.53 |
| LD_1_1 | 45200295 | 43323860 | 4.33G | 0.03 | 97.06 | 90.81 | 49.17 |
| LD_1_2 | 45200295 | 43323860 | 4.33G | 0.04 | 95.65 | 88.66 | 49.28 |
| LD_2_1 | 35742634 | 33203394 | 3.32G | 0.04 | 96.42 | 89.25 | 48.79 |
| LD_2_2 | 35742634 | 33203394 | 3.32G | 0.04 | 95.26 | 86.82 | 48.86 |
| LD_3_1 | 41248743 | 38141174 | 3.81G | 0.04 | 96.62 | 89.54 | 48.93 |
| LD_3_2 | 41248743 | 38141174 | 3.81G | 0.05 | 94.88 | 86.07 | 48.98 |

Note: ^a^HP means cows with high milk protein percentage at peak lactation, LP means cows with low milk protein percentage at peak lactation, HD means cows with high milk protein percentage under non-lactating period, LD means cows with low milk protein percentage under non-lactating period. HP1, HP2 and HP3 refer to the three biological replicates cows in HP group, the rest of groups are with same name rules. HP_1_1 and HP_1_2 mean the paired-end reads for the same individual using paired-end sequencing, others are same.

^b^Q20: **the proportion of bases with a** phred base quality score greater than 20; i.e., the proportion of read bases whose error rate is less than 1%.

^c^Q30: **the proportion of bases with a** phred base quality score greater than 30; i.e., the proportion of read bases whose error rate is less than 0.1%.

**Supplementary Table S2**. Summary of sequence read alignments to the reference genome

| Sample Name | HP1 | HP2 | HP3 | LP1 | LP2 | LP3 |
| --- | --- | --- | --- | --- | --- | --- |
| Total reads | 72154292 | 88016924 | 77449682 | 75585878 | 75995344 | 69356272 |
| Total mapped | 66820805 (92.61%) | 81849590 (92.99%) | 71728714 (92.61%) | 69478072 (91.92%) | 70765310 (93.12%) | 64601124 (93.14%) |
| Multiple mapped | 2027757 (2.81%) | 2072433 (2.35%) | 2627485 (3.39%) | 2485498 (3.29%) | 2473615 (3.25%) | 1688509 (2.43%) |
| Uniquely mapped | 64793048 (89.8%) | 79777157 (90.64%) | 69101229 (89.22%) | 66992574 (88.63%) | 68291695 (89.86%) | 62912615 (90.71%) |
| Read-1 | 32727756 (45.36%) | 40236273 (45.71%) | 34989813 (45.18%) | 33973186 (44.95%) | 34486953 (45.38%) | 31787116 (45.83%) |
| Read-2 | 32065292 (44.44%) | 39540884 (44.92%) | 34111416 (44.04%) | 33019388 (43.68%) | 33804742 (44.48%) | 31125499 (44.88%) |
| Reads map to ‘+’ | 32213866 (44.65%) | 39872467 (45.3%) | 34538777 (44.6%) | 33400332 (44.19%) | 34161724 (44.95%) | 31398060 (45.27%) |
| Reads map to ‘-’ | 32579182 (45.15%) | 39904690 (45.34%) | 34562452 (44.63%) | 33592242 (44.44%) | 34129971 (44.91%) | 31514555 (45.44%) |
| Non-splice reads | 31742273 (43.99%) | 39785504 (45.2%) | 34246525 (44.22%) | 33096238 (43.79%) | 35188476 (46.3%) | 30205386 (43.55%) |
| Splice reads | 33050775 (45.81%) | 39991653 (45.44%) | 34854704 (45%) | 33896336 (44.84%) | 33103219 (43.56%) | 32707229 (47.16%) |

Note: Bovine genome **UMD3.1 (**<http://www.ncbi.nlm.nih.gov/genome/guide/cow/index.html>**)** was used for the alignment.

**Continued Table S2**:

| Sample Name | HD1 | HD2 | HD3 | LD1 | LD2 | LD3 |
| --- | --- | --- | --- | --- | --- | --- |
| Total reads | 67911260 | 78336492 | 79164622 | 86647720 | 66406788 | 76282348 |
| Total mapped | 61952067 (91.23%) | 71420786 (91.17%) | 72278425 (91.3%) | 79933351 (92.25%) | 60128914 (90.55%) | 69013352 (90.47%) |
| Multiple mapped | 1099237 (1.62%) | 1141797 (1.46%) | 1262864 (1.6%) | 1719645 (1.98%) | 1121466 (1.69%) | 1200422 (1.57%) |
| Uniquely mapped | 60852830 (89.61%) | 70278989 (89.71%) | 71015561 (89.71%) | 78213706 (90.27%) | 59007448 (88.86%) | 67812930 (88.9%) |
| Read-1 | 30724245 (45.24%) | 35407163 (45.2%) | 35705880 (45.1%) | 39458174 (45.54%) | 29848966 (44.95%) | 34406028 (45.1%) |
| Read-2 | 30128585 (44.36%) | 34871826 (44.52%) | 35309681 (44.6%) | 38755532 (44.73%) | 29158482 (43.91%) | 33406902 (43.79%) |
| Reads map to ‘+’ | 30424274 (44.8%) | 35142878 (44.86%) | 35511847 (44.86%) | 39117700 (45.15%) | 29499092 (44.42%) | 33895871 (44.43%) |
| Reads map to ‘-’ | 30428556 (44.81%) | 35136111 (44.85%) | 35503714 (44.85%) | 39096006 (45.12%) | 29508356 (44.44%) | 33917059 (44.46%) |
| Non-splice reads | 42923546 (63.21%) | 50676700 (64.69%) | 49618443 (62.68%) | 55779195 (64.37%) | 42229789 (63.59%) | 48323166 (63.35%) |
| Splice reads | 17929284 (26.4%) | 19602289 (25.02%) | 21397118 (27.03%) | 22434511 (25.89%) | 16777659 (25.26%) | 19489764 (25.55%) |

Note: Bovine genome **UMD3.1 (**<http://www.ncbi.nlm.nih.gov/genome/guide/cow/index.html>**)** was used for the alignment.

**Supplementary Table S3. RNA-Seq gene expression results in mammary tissues of all 12 Chinese Holstein cows**

| RPKM Interval | HP1 | HP2 | HP3 | LP1 | LP2 | LP3 |
| --- | --- | --- | --- | --- | --- | --- |
| 0~1 | 17321(62.88%) | 17377(63.09%) | 16836(61.12%) | 16339(59.32%) | 15824(57.45%) | 17182(62.38%) |
| 1~3 | 4028(14.62%) | 3827(13.89%) | 4001(14.53%) | 3906(14.18%) | 3823(13.88%) | 3812(13.84%) |
| 3~15 | 4797(17.42%) | 4778(17.35%) | 5156(18.72%) | 5524(20.06%) | 5954(21.62%) | 4982(18.09%) |
| 15~60 | 1098(3.99%) | 1213(4.40%) | 1217(4.42%) | 1389(5.04%) | 1549(5.62%) | 1218(4.42%) |
| > 60 | 300(1.09%) | 349(1.27%) | 334(1.21%) | 386(1.40%) | 394(1.43%) | 350(1.27%) |

Note: RPKM means reads per kilobase of transcriptome per million mapped reads, indicating the gene expression level.

**Continued Table S3**:

| RPKM Interval | HD1 | HD2 | HD3 | LD1 | LD2 | LD3 |
| --- | --- | --- | --- | --- | --- | --- |
| 0~1 | 11938(43.34%) | 11993(43.54%) | 12122(44.01%) | 12213(44.34%) | 12025(43.66%) | 12092(43.90%) |
| 1~3 | 2682(9.74%) | 2590(9.40%) | 2583(9.38%) | 2525(9.17%) | 2686(9.75%) | 2642(9.59%) |
| 3~15 | 6078(22.07%) | 6079(22.07%) | 6090(22.11%) | 6038(21.92%) | 6215(22.56%) | 6141(22.30%) |
| 15~60 | 5419(19.67%) | 5441(19.75%) | 5312(19.29%) | 5255(19.08%) | 5129(18.62%) | 5224(18.97%) |
| > 60 | 1427(5.18%) | 1441(5.23%) | 1437(5.22%) | 1513(5.49%) | 1489(5.41%) | 1445(5.25%) |

Note: Same to the above.

**Supplementary Table S10. PCR primers for qRT-PCR validation of 19 differentially expressed genes between the mammary tissue with high milk protein percentage and with low milk protein percentage during the peak and dry lactation.**

| Gene name | Log_2_Fold change  by RNA-seq | Forward primer sequence | Reverse primer Sequence | Start bp | Stop bp | Amplicon (bp) | Tm (°C) |
| --- | --- | --- | --- | --- | --- | --- | --- |
| *ANG* | −0.85 | GGAAGAAGAAGCGGGTGAGAA | GACCATGACCATCTCTTCCAACA | 43 | 147 | 105 | 58 |
| *CD14* | −1.03 | GCGTGAGCCACTGTAAAGGAA | GTAGGGCACGCACACCATAGT | 1 | 100 | 100 | 60 |
| *CLU* | 1.34 | TGGGCAATCTCGGACAAAG | TCACCTCCTTGAGGGCATTT | 121 | 211 | 91 | 57 |
| *CNTFR* | −0.95 | TCCCACCTACATCCCCAACA | GATGTGGCAGCGGTTCTTG | 681 | 780 | 100 | 60 |
| *CYP1A1* | −1.20 | CAGCCTGATTGAGCACTGTCA | TCAAACCCGGCTCCAAAG | 1313 | 1423 | 111 | 58 |
| *ENPP5* | 1.05 | TTGAGTTGGCGAGCAGATTATC | TGAAAGTGTTAATGCAGCAAGCA | 7 | 111 | 105 | 55 |
| *ERBB2* | 1.00 | TCTGGAGGAGATCACAGGTTACC | CGAGTAGGCACCATCATGCA | 1430 | 1550 | 121 | 61 |
| *FCGR3A* | −1.15 | CGGGTCTAAAAACGAGTCTTCAG | AAGGTGATCTGGTGCCAAGGT | 297 | 401 | 105 | 57 |
| *GALE* | −0.74 | GTGCTGCTGCGGTATTTCAA | ATTGCCACCTGGGAGACGTA | 691 | 800 | 110 | 61 |
| *HSPA8* | −1.13 | CGAATCATCAATGAGCCAACTG | TGCCACCCCCTAAATCAAAG | 586 | 685 | 100 | 57 |
| *LPAR6* | 0.85 | CCTTGTATGGGTGCATGTTCAG | TTGTTTCATTCCGCACTTTGAG | 582 | 686 | 105 | 56 |
| *LTF* | 1.26 | CCCTATGGGAATCCTTCGCC | CAGTTGACACAGGTTGGGGT | 577 | 712 | 136 | 59 |
| *MAFB* | -1.21 | ACCAGGGTGTGCAGAGTTTCA | CTGCTTTCCCTCCTTTTCTTGTTAG | 2483 | 2587 | 105 | 60 |
| *MEGF10* | -0.85 | AGCTTGGTGCTTTTGGACTTGA | GGCGTATGGGTTTTCAGAACTG | 3119 | 3237 | 119 | 57 |
| *NARS* | -0.57 | TTTGCATCGCACAGTCATACAG | TCGAAGGTCAGGAAAGGACACT | 1052 | 1151 | 100 | 60 |
| *NEDD4L* | 1.16 | ATGAATGGATTTGCCGAGCTT | GTGTGAGCTCTGGGCAGTTTT | 2894 | 2988 | 95 | 57 |
| *RRM2* | 1.79 | GCGTCGATATTCTGGCTCAAG | GAACATCAGGCAGGCAAAGTC | 772 | 882 | 111 | 58 |
| *SERPINA1* | 2.51 | ACAGTTTTTGCTCTGGTGAATTACA | TGCTCGTCCACATGGAAGTC | 635 | 735 | 101 | 57 |
| *UBE2C* | 1.89 | TGATAGCCCTTTGAACACACATG | TTGGCTGGAGACCTGCTTTG | 485 | 584 | 100 | 58 |
| *GAPDH* |  | AATGGAAAGGCCATCACCATC | GTGGTTCACGCCCATCACA | 273 | 476 | 204 | 59 |
| *MARVELD1* |  | GGCCAGCTGTAAGATCATCACA | TCTGATCACAGACAGAGCACCAT | 2262 | 2361 | 100 | 59 |

**Supplementary Figure S1. Relative mRNA abundances in the mammary tissues at peak lactation**

**
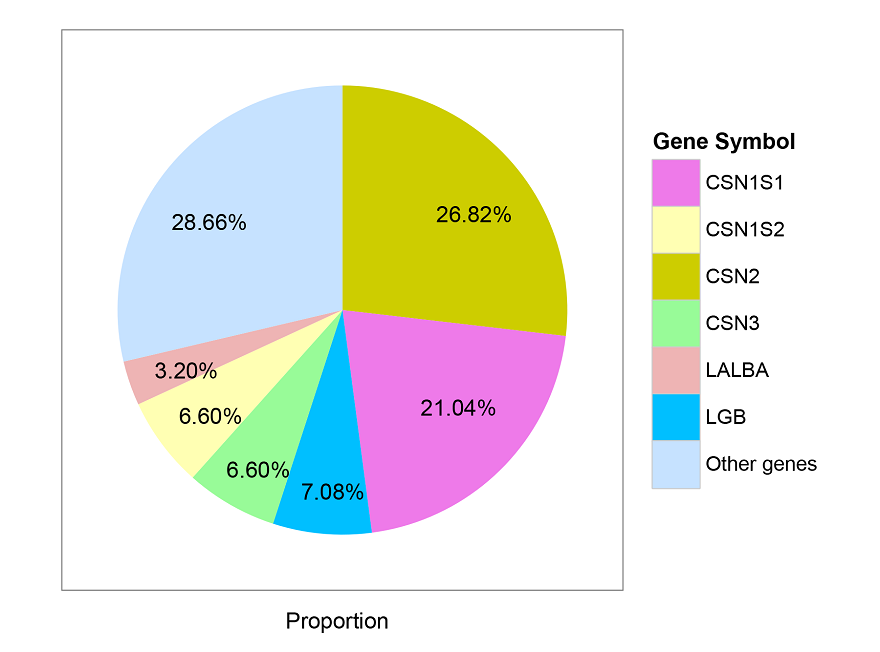
**

**Supplementary Figure S2. The comparisons of expression abundance of 19 randomly differential expressed genes between qRT-PCR and RNA-Seq**


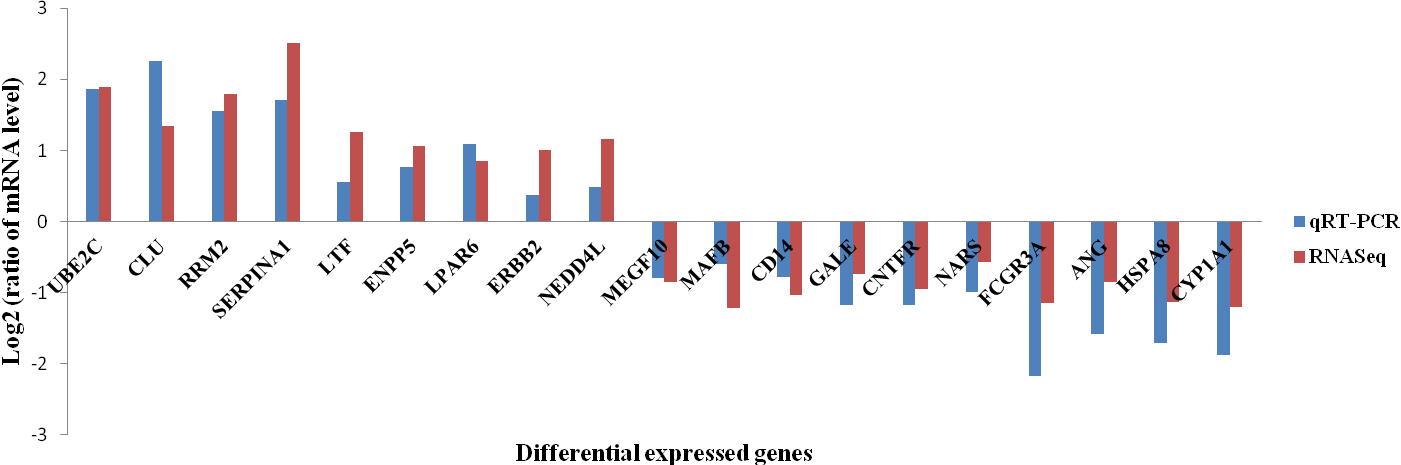


**Supplementary Figure S3. The statistical power of experimental design**


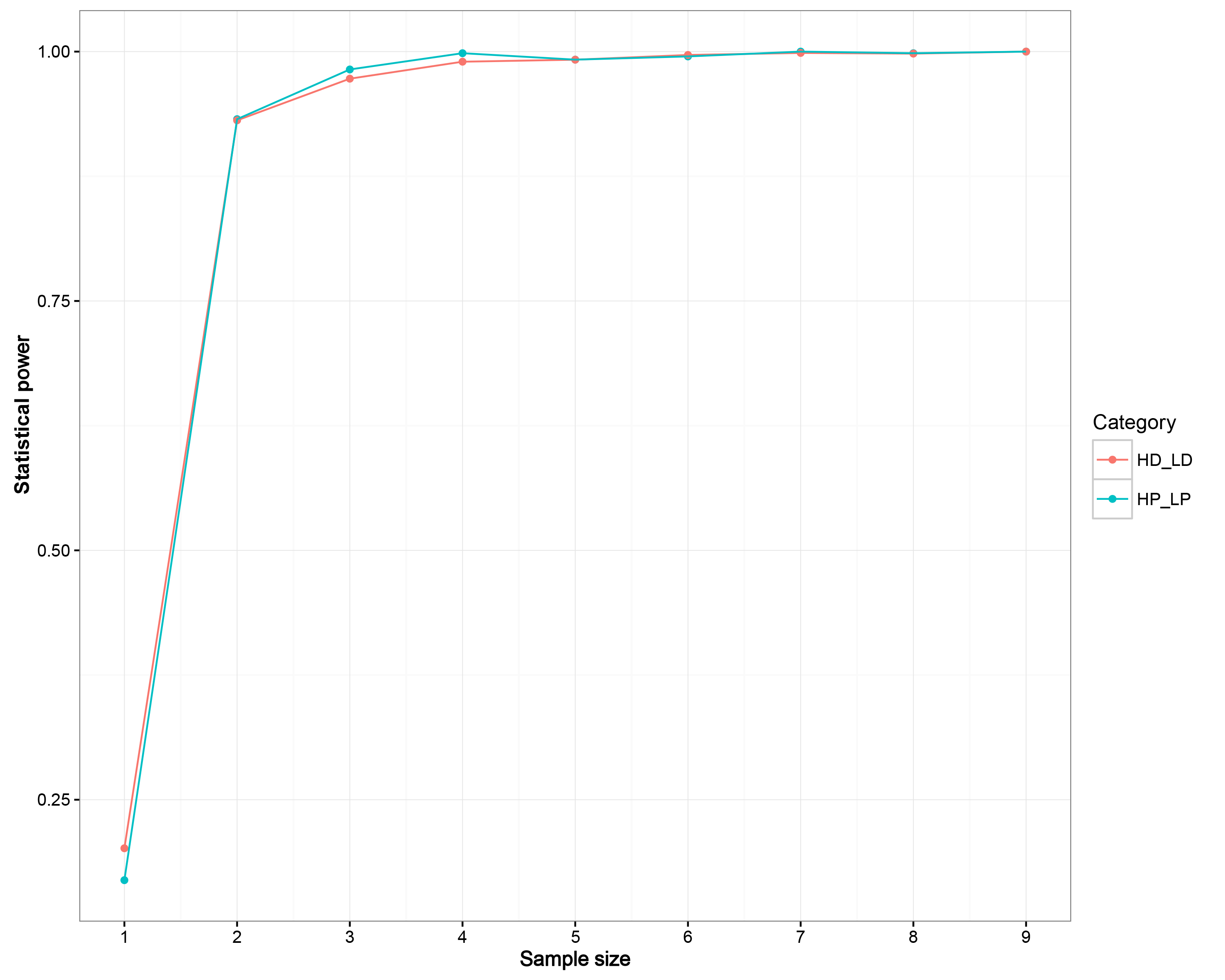


**Legends:** The y-axis refers to statistical power of experimental design; the x-axis refers to the sample size of the biological replicates in each treatment.
